# Supplementary figures and images for: Auranofin Resistance in Toxoplasma gondii Decreases the Accumulation of Reactive Oxygen Species but Does Not Target Parasite Thioredoxin Reductase
Source: Front Cell Infect Microbiol. 2021 Mar 19;11:618994. doi: 10.3389/fcimb.2021.618994 (PMC8017268; doi:10.3389/fcimb.2021.618994)

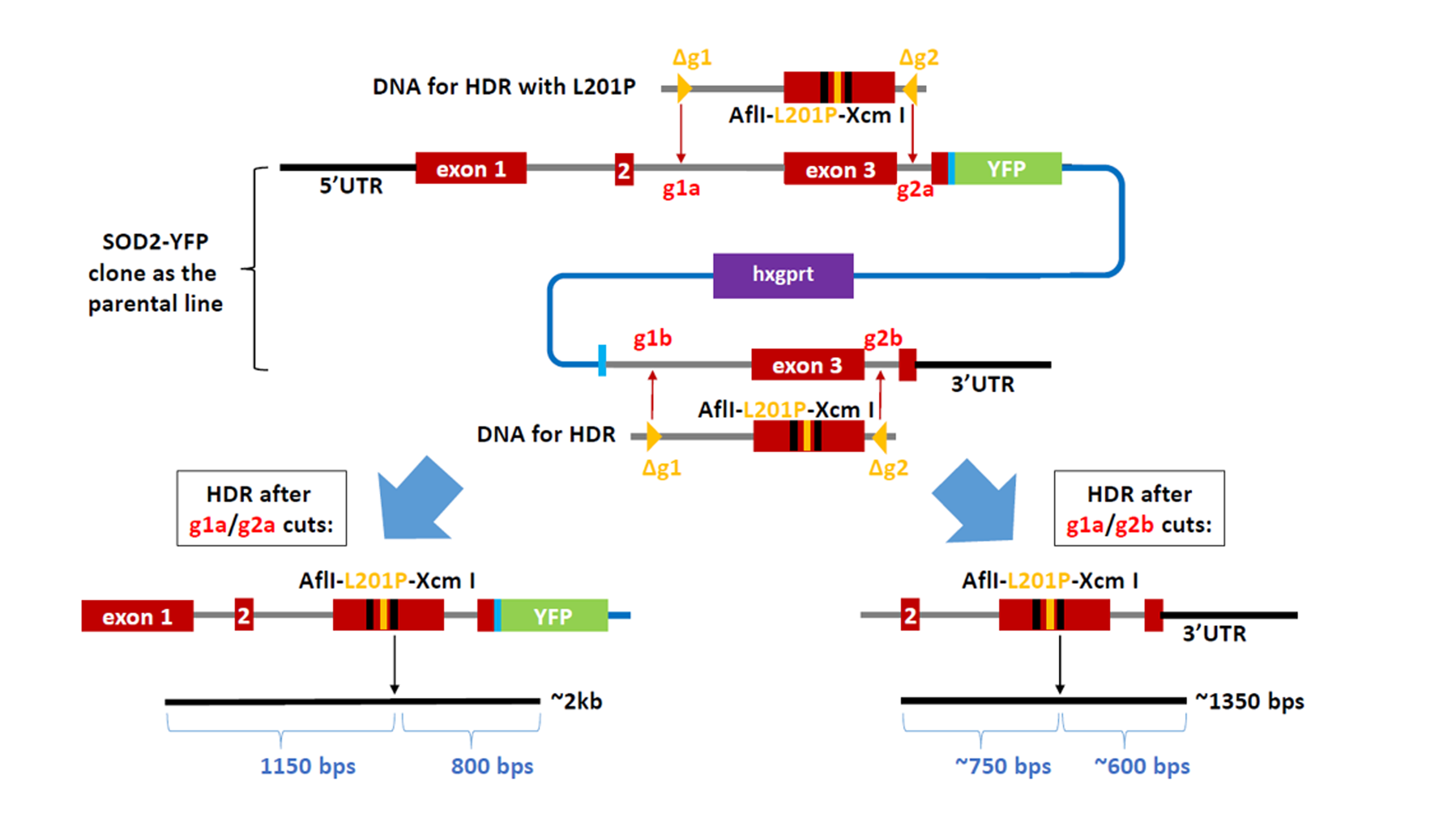

Supplement: Supplementary Figure 1 — CRISPR-Cas9 strategy for generating the T. gondii SOD2.L201P line. TgSOD2-YFP parasites incorporating the LIC plasmid (blue line) undergo CRISPR-Cas9 mediated homologous directed repair (HDR). The DNA amplicon used for HDR contains the L201P mutation (gold vertical line in exon 3) and two silent and unique restriction sites AflI and XcmI (black vertical lines in exon 3), which allow for screening of positive clones. Sequences corresponding to the two gRNA were deleted from the DNA for HDR (Δg1 and Δg2, golden triangles). Each of the gRNA was present twice in TgSDO2-YFP parasites (g1a/b, g2a/b; red font), resulting in two T. gondii SOD2.L201P lines. For HDR after g1a/g2a cuts, a T. gondii SOD2.YFP.L201P line is generated (lower left). For HDR after g1a/g2b cuts, a T. gondii SOD2.L201P line without YFP or LIC plasmid (blue) is generated (lower right). Positive clones were screened with XcmI digest before sequence verification. [file Image_1.tif]

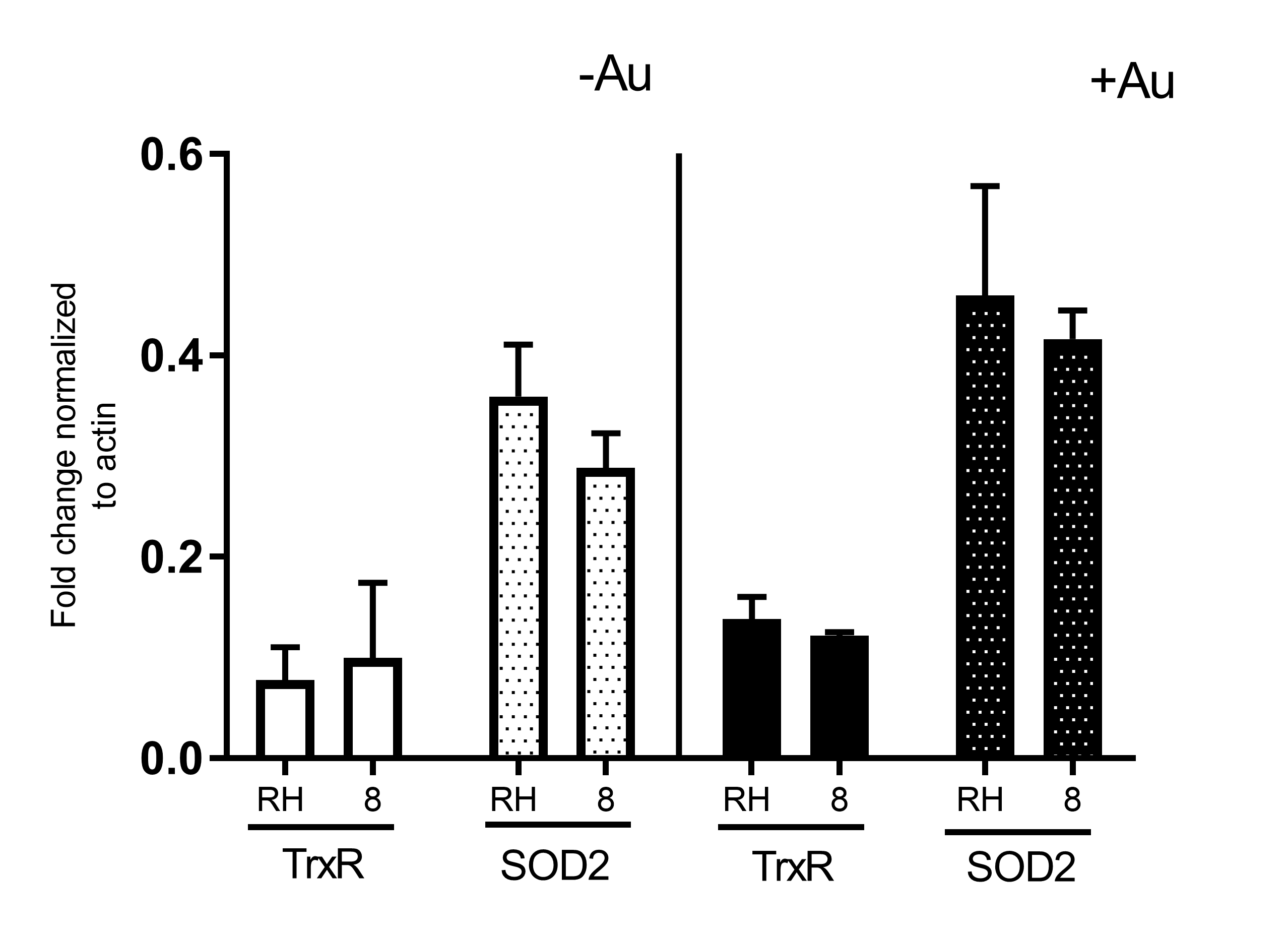

Supplement: Supplementary Figure 2 — Auranofin treatment does not change the expression of thioredoxin reductase nor superoxide dismutase 2 transcripts. RNA was harvested from freshly lysed parasites incubated in either control or 1µM auranofin containing media. Relative expression of SOD2 and TrxR genes normalized to actin. Parasites with or without exposure to auranofin, expressed similar transcripts amounts of TrxR and SOD2 (p>0.05, Mann-Whitney U-Test). There were no statistical differences. This manuscript has been released as a pre-print at https://www.biorxiv.org/as (Ma C et al., 2020). [file Image_2.tif]
