# Supplementary material for: Auranofin Resistance in Toxoplasma gondii Decreases the Accumulation of Reactive Oxygen Species but Does Not Target Parasite Thioredoxin Reductase
Source: Front Cell Infect Microbiol. 2021 Mar 19;11:618994. doi: 10.3389/fcimb.2021.618994 (PMC8017268; doi:10.3389/fcimb.2021.618994)
Supplement: Supplementary Table 1 — Primers for SNV validation. [file Table_1.pdf]

Name	Gene ID*	Sequence	Function
TrxR 12F	309730	GGACCTATGAGTTGCCTCTCTG	TrxR amplification 5' half
TrxR R10	309730	GTCAAATCCCAATTCGCGGAG	TrxR amplification, 5' half
TrxR For 8	309730	GTCTGCTCTCTCTGTTTCGCAAG	TrxR amplification, 3' half
TrxR Rev 9	309730	CCTCCACATCCTCCAGAAGC	TrxR amplification, 3' half
5TrxR 1R	309730	CGAGTTTTCTAGAGCGTGTGC	TrxR sequencing, 5' half, exon 1
5TrxR 1F	309730	CATCCCTGTGCACTTTCC	TrxR sequencing, 5' half, exons 2-3
5TrxR 3F	309730	GTGGATACCCTCGTATAATATGC	TrxR sequencing, 5' half, exons 4-5
TrxR For 8	309730	GTCTGCTCTCTCTGTTTCGCAAG	TrxR sequencing, 3' half, exons 6-7
3TrxR 2F	309730	GCTTCGTTTGATTGCCTGTC	TrxR sequencing, 3' half, exons 8-9
3TrxR 3F	309730	CATCTTGTTCCTCCAACCTCTG	TrxR sequencing, 3' half, exon 10
3TrxR 4F	309730	GATGTCTGCTTTCGTCTTCC	TrxR sequencing, 3' half, exon 11
SOD LIC3	316330	TACTTCCAATCCAATTTACGAAGGTGAGGAGCATTCG	SOD2 amplification with LIC casette
SOD LIC5	316330	TCCTCCACTTCCAATTTTAGCGTTGCTTTCAAGTGCTTTCACC	SOD2 amplification with LIC casette
SOD2 RE Ins F	316330	TGATAAGCTAGCCAGTGATCT	Site directed mutagenesis for NheI
SOD2 RE Ins R	316330	TCTCCATCTCCCCCTGAT	Site directed mutagenesis for NheI
SOD2 SNP F	316330	TTTTCCAAACCAGCGGCAGGC	Site directed mutagenesis for L201P
SOD2 SNP R	316330	CTCATCCTTGAACTGGGC	Site directed mutagenesis for L201P
SOD Seq1	316330	CTTTCGAATCCTGAAACACC	Validation of SOD2 SNV
SOD Seq2	316330	GTCATGTGTGTTTGCCCCGTAG	Validation of SOD2 SNV
SOD2 F	316330	GCAGAGACTCTTCGCTTTCAC	Amplification of SOD2.YFP CRISPR clone
AmpR	N/A	CCTTGAAGAAGATGGTGCGC	Amplification of SOD2.YFP CRISPR clone
SOD E2	316330	GCTTCATTCAAGGCACACC	Amplification of SOD2 in CRISPR clone
SOD R	316330	CTATCCAGGGTTCACGACG	Amplification of SOD2 in CRISPR clone
CDS F	300120	GTGTGTCTGCATCGTTTCC	Aminotransferase amplification
CDS R	300120	GTTCCTCTGAAGCATGTTTCC	Aminotransferase amplification
CDS E1	300120	CTCTGTTTGGTGTCTTTGACC	Aminotransferase SNV sequencing
COR F	253120	CTCAGCAATGTGACATGCTAGC	Mandelonitrile lysase amplification
COR R	253120	GTTCGACGGTGAAATGTGCTC	Mandelonitrile lysase amplification
COR E8	253120	CTTGCTACCATTTCTCCTCG	Mandelonitrile lysase SNV
MBL F	234250	GTGAACTCAGTGAAACCGC	MAEBL amplification

MBL R	234250	GCACTCTCGAGTTTTACGC	MAEBL amplification
MBL E2	234250	GGAGGATCAGGTTTATGAGTGC	MAEBL SNV sequencing
NAD F	244700	CGTCTCCTTCTCTCGATGG	NAD(+)/NADH kinase amplification
NAD R	244700	CGTGAACTTTAATGGATGGCG	NAD(+)/NADH kinase amplification
NAD E7	244700	CTTCCCAGTTCTTTCTCTCC	NAD(+)/NADH kinase SNV
SOD F	316330	CCTCTCGAGCAGTTTACTCG	SOD2 amplification
SOD R	316330	CTATCCAGGGTTCACGACG	SOD2 amplification
SOD E2	316330	GCTTCATTCAAGGCACACC	SOD2 SNV sequencing
SOD E1	316330	CATCTTCGCGTTTCCTGC	SOD2 SNV sequencing
SWI F	245720	GTTTCCTCGTCTGTGCAC	SWI2/SNF2 amplification
SWI R	245720	GTTATTCACTATCCCAGGCAC	SWI2/SNF2 amplification
SWI E2	245720	GTGTGTGTCAAGAGGTCG	SWI2/SNF2 SNV sequencing
TPR F	268870	CCGAGAAAGTGAATTGCC	Tetratricopeptide amplification
TPR R	268870	CACACACACACAGAAACG	Tetratricopeptide amplification
TPR E4-2	268870	CCAACTGTTCCGGTGTCTC	Tetratricopeptide SNV sequencing
ZnC F	311100	CCTGCATTCTACAATAGGCGC	Zn finger CCCH type amplification
ZnC R	311100	GTTTCAGAAGCAGCTCCTGG	Zn finger CCCH type amplification
ZnC 4F	311100	CAAGCACGCCAATCACTAG	Zn finger CCCH type SNV

*Gene ID # in Table 1.
